# The Improved Image Scrambling Algorithm for the Wireless Image Transmission Systems of UAVs

**DOI:** 10.3390/s18103430

**Published:** 2018-10-12

**Authors:** Jie Dong, Guowei Wu, Tingting Yang, Yangyang Li

**Affiliations:** 1 School of Software, Dalian University of Technology, Road No. 8, Dalian 116620, China; dj_0609@126.com (J.D.); wgwdut@dlut.edu.cn (G.W.); 2 School of Navigation, Dalian Maritime University, Dalian 116620, China; lyy218216@163.com

**Keywords:** UAV, high-definition long-distance wireless digital image transmission system, improved image scrambling encryption algorithm, Fibonacci-*p* coding

## Abstract

With the deepening of modern military reforms, information has become the key to winning modern warfare. The use of unmanned aerial vehicle (UAV) to capture image information has become an important means of reconnaissance in modern warfare and plays an irreplaceable role. The image information usually uses a wireless image transmission system, since image information is intercepted or stolen easily during the information transmission, encrypting an image is a common method for ensuring image security. However, traditional encryption algorithms have some deficiencies in terms of efficiency and security. In order to overcome these shortcomings, a new algorithm is proposed in this paper-an improved image scrambling encryption algorithm based on Fibonacci-*p* coding. The first new idea of the algorithm is to separate the positive and negative signs and data of the scrambled DCT coefficients, then form the symbol matrix and the data matrix respectively, perform the scrambling encryption operation on the symbol matrix. The second new idea is to encrypt the color RGB image by converting the *R*, *G*, and *B* colors into *Y*, *Cb*, and *Cr*, and converting the normal image operation into operations on *Y*, *Cb*, and *Cr*, thereby implementing the encryption operation. The comprehensive performance of the algorithm is optimal with different image information. Experiments results validate the favorable performance of the proposed improved encryption algorithm.

## 1. Introduction

The biggest advantage of UAVs in combat operations has always been reflected in intelligence reconnaissance. The reconnaissance images obtained by the UAV loaded with various imaging sensors must be transmitted to the commanders in real time to make the battlefield transparent. Therefore, the transmission of reconnaissance information is a major aspect of the UAV system and an important part of the UAV measurement and control system. Video and flight data collected by various types of drones are usually transmitted using a wireless image transmission system. Compared with wired image transmission, wireless image transmission has great advantages in terms of mobility and power consumption, its real-time performance and image transmission speed and quality are much lower than wired image transmission: (1) the image transmission clarity is not enough and the transmission distance is close and the application requirements cannot be fulfilled; (2) the communication corridor has no communication link, and the transmission line patrol video cannot be transmitted back to the monitoring center in real time; (3) patrol data processing, patrol pictures, video analysis, naming, archiving, and query are all manual operations, with low intelligence and low efficiency. Especially the patrol video and the live tower number cannot automatically correspond, rely on manual operations mainly. Therefore, in connection with the development of drone inspection technology, high-definition long-distance wireless digital image transmission system has far-reaching significance.

Image scrambling technology is a common method of image encryption, and is often used as a preprocessing technique for digital watermarking. In the field of image scrambling, many experts and scholars at home and abroad have proposed different theoretical systems [1], the purpose of which is to provide safer encryption methods. The purpose of image scrambling is to make the encrypted image and the original image statistical information completely different by a certain transformation algorithm, thereby conserving the real content of the original image. The image scrambling schemes that have been summarized can be divided into three categories: (1) space-based scrambling algorithm; (2) color space-based scrambling algorithm [2]; (3) scrambling algorithm based on position and color space. For the first type of program, experts have proposed many classical algorithms, such as Arnold transform, magic square transform, Fibonacci transform, panel changer, Hilbert curve method, Tangram algorithm, IFS model, chaos and so on. This kind of algorithm does not change the gray value of the image, no matter how exquisite the algorithm is designed, it can’t change the basic statistical features of the image (such as histogram), which is easy to attract the attention of the attacker. For the scrambling of the image color space, the classic Gray Code conversion is often used. Hu designed a scrambling transformation system based on Logistic and standard mapping, which changes the image pixel values and generates mutual dependence by alternative transformation [3], so the encryption algorithm has the basic elements of encryption system such as scrambling, substitution and diffusion. The second type of scheme changes the statistical information of digital images greatly, but the algorithm itself has limitations, such as the encryption effect is not good, and the application is not wide, compared with the first two types of schemes, the third scheme of the system scheme expands the algorithm. The key space combines pixel location scrambling and pixel value scrambling to compensate for the mutual defect that is the development direction of image scrambling.

In this paper, according to specific requirements of the wireless image transmission system and combining with the research progress of image encryption technology that an improved image scrambling encryption algorithm based on Fibonacci-*p* coding is used. The algorithm combines with the process of video compression and regulates the parameters of the encryption process that according to the content of the video image, so that the comprehensive performance of the algorithm is optimal for different image information. The results of the experiment that the algorithm has good performance in the influence of adjacent pixels, and the information entropy of images is more ideal than other algorithms. This work targets to investigate the security issues in military communication system, which is featured by protecting important data. Specifically, the contributions of this paper are three-fold:

(1) We propose a high-definition long-distance wireless image transmission system to transmit image information to the command center in real time in order to make the battlefield transparent.
(2) A novel algorithm namely improved image scrambling encryption algorithm based on Fibonacci-*p* coding is proposed, which has optimal comprehensive performance with respect to the security issue.
(3) It has been proved from the experimental results that the superior performance of the proposed improved image scrambling encryption algorithm.

The rest of the paper is organized as follows: the Materials and Methods are presented in Section 2. The system model is presented in Section 3. Section 4 presents the improved image encryption algorithm. Section 5 provides the experimental results and related analysis. Finally, we conclude our main work in Section 6.

## 2. Materials and Methods

There are two main concepts in wireless image transmission that is real-time video transmission. One is mobile transmission that is mobile communication, and the other is broadband transmission that is broadband communication. In the past, wireless image transmission was mainly based on one-way analog TV broadcasting service [4,5,6,7,8,9]. Mainly, a set of TV programs use a single frequency point. The single frequency network can improve the utilization of frequency resources. However, when TV programs are broadcast at the same frequency and at the same frequency in different places, they will interfere with each other. In addition, Since the receiving or transmitting party is in a moving state, whether it is transmitting or receiving, it will encounter strong multipath interference, that is, echo interference. Therefore, the processing of echo interference may fundamentally affect the real-time transmission of a wireless high-definition video. The performance of the system, and the COFDM transmission technology in the VFORD8000 wireless digital high-definition video real-time transmission system is the problem that can effectively use the echo instead of negatively eliminating the echo.

In image encryption technology, digital image scrambling technology is very important. In general, the better the effect of image scrambling, as the secret information hidden in the carrier information, the stronger the anti-detection capability, the higher the hiding ability. At present, the research on scrambling encryption algorithms for grayscale images has made great progress. Nowadays, the image scrambling algorithms commonly used by researchers include Arnold transform, Hilbert transform, magic square transform, Fibonacci transform and affine transform. Digital image scrambling technology only scrambles the image space at the beginning, but today, for digital images, only scrambling the image position can’t meet our needs, so people continue to study on this basis and find. In addition to scrambling in the spatial domain of the digital image (including color space and location space), the scrambling process can also be performed in the frequency domain of the digital image. This discovery has led to further improvements in the implementation of digital image technology. As one of the digital image encryption methods, digital image scrambling technology allows legal users to freely control parameter selection, algorithm selection and random number technology to improve the difficulty of attackers and illegal attacks. For the spatial disturbance of the image, it just uses an algorithm to destroy the order of the individual pixel positions of the image, making an image “unrecognizable”, in fact the total number of pixels is unchanged, the histogram is also unchanged, and the transformation The latter image does not reflect the information of the original image, so even if the image information is illegally acquired, the acquirer has no way to directly obtain valuable information from it. For example, Lu and Sun proposed an image encryption algorithm based on four-dimensional chaos system [10]. Penvy at al. proposed an LSB matching detection algorithm that implements image encryption using a method of eliminating pixel adjacency matrix (SPAM) [11]. Monga and Mihcak proposed a safe and robust image hashing algorithm by non-negative matrix factorization, which changes the pixel position of the image and changes the pixel value of the image [12].

We study image scrambling technology mainly for two purposes: the first purpose is to encrypt the image so that it is difficult to restore the previous image without knowing the algorithm used for scrambling. The second is scrambling technology as a preprocessing method for information hiding to enhance the robustness of image processing. Networks put everybody in contact and collect image information. They have been applied to all aspects of human social life as a large-capacity information carrier. The technique of scrambling digital images is one of the most important image encryption measures that have been studied in recent years. This image encryption technology is independent, and its scrambling method does not generate redundant information, which is especially suitable. Combine large images for encryption. The digital image processing technology can be that the visual effect of the image presents a random distribution of each pixel, and the image information is very well encrypted, thereby improving the security of image transmission to a certain extent. Image scrambling is used as a pre-processing of information hiding, interfering with the grayscale distribution of secret information, making it more like noise added to image files. The scrambling of information is not only conducive to concealment, but also plays a very important role in covert communication in terms of covert communication against attacks against, detection and imperceptibility of secret information.

## 3. System Model

**Figure 1:**
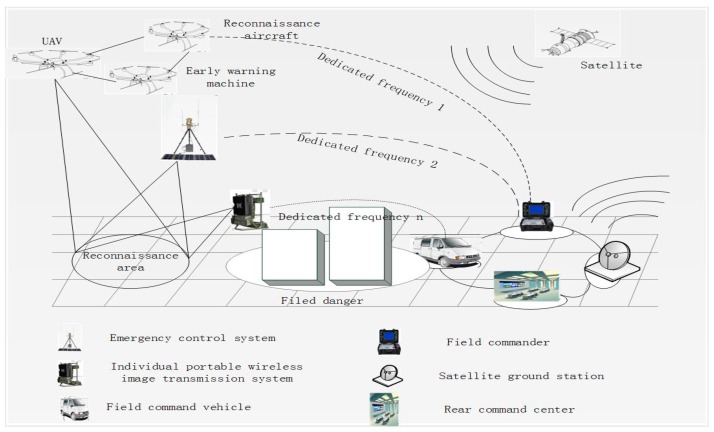
Information Transmission Diagram.

Unmanned aerial vehicles are an indispensable part of modern warfare. Together with spy satellites, reconnaissance planes and early warning planes they make up a modern investigation network (Figure 1). With its unmanned, low-cost and flexible maneuvering features, the UAV has replaced the large-scale reconnaissance aircraft as the most important investigative force on the frontier of the battlefield. As an advanced remote sensing data acquisition method, UAV reconnaissance has been used in military reconnaissance widely, target surveillance, damage assessment, map mapping, land surveying, disaster monitoring, meteorological detection, etc. Resulting in huge economic, military and social benefits and show a wide range of application prospects. Designed airborne module, integrated the camera and the wireless transmitting module to form a camera with wireless transmission function, the camera is divided into high-definition video capture and compression and encryption, and the video capture module adopts High-end movement that the functions such as automatic glare suppression and high-speed auto focus. By adopting automatic glare suppression and auto focus movement, the airborne module can perform video capture efficiently and obtain camera focal length information in real time; the video compression module adopts H.265 hard compression method, compared with other methods, the time required less, and the effect is better, the video encryption use image scrambling encryption algorithm based on Fibonacci-*p* coding. The wireless transmitting module consists of information modulation and power amplifier. The wireless receiving part consists of a buffer and demodulation, decoding, and display modules. More importantly, it can complete high quality video and transfer of focal length information up to 5 km.

The overall scheme of wireless transmission is roughly divided into the following five blocks: (1) video acquisition; (2) video codec; (3) system core control; (4) radio frequency communication. The video capture part function is to complete the collection of video information, collect external video information through CMOS image sensor chip and convert it into electric signal for video encoding processing; the codec part is used for signal encoding of high definition video and decoding of receiving end mainly; The function of system core control part is to control the acquisition, transmission and reception of the video signal; the radio frequency communication part transmits and receives the high-definition video by directly transmitting and receiving the electromagnetic wave signal to achieve the purpose of communication.

### 3.1. Video Capture Section

The video data acquisition module is the initial input of information, and its performance is crucial to the whole system. The quality of the original video image will determine the image clarity that can be displayed at the headquarters command center. This section uses the GoPro outdoor sports satellite camera and corresponding control components. The monitoring content includes the situation at the time of a battle, the battlefield situation after a war ends, and the reconnaissance situation before a war begins.

### 3.2. Video Coding Part

The compression and encryption modules of the video data are the core of the wireless image transmission system. The performance of the module directly affects the overall performance of the entire system. The compression of video data is limited by multiple conditions, such as the bandwidth of the network, the resolution and frame rate of the image, the maximum delay of the code, and the performance of the processor. Therefore, the video compression module needs to be coordinated with the transmission module. At the same time, since the encryption process is integrated into the process of compression, the relationship between the two is very close and affects each other. The video codec part of the system uses the Hai Si Hi3516A video codec chip, which is implemented by the industry’s latest H.265 video compression encoder and Secure Core processor with DSP core, while adopting advanced low-power process. And low-power architecture design, they will make the Hi3516A have a huge advantage in its class, not only has a low bit rate, and excellent image clarity, at the same time low power is also an important reason to choose it.

### 3.3. System Core Control Part

The core control part of the system controls the acquisition, transmission and reception of video signals. OMAP5910 is used as the main control chip of the system to receive other module information and send command control to other module functions. The dual-core application processor OMAP5910 enables multiple interconnects, embedded, remote, and many other applications. Among them, TI’s used TMS320C55XDSP can be used for real-time multimedia, while the fully functional ARM925 can meet control and interface demands.

### 3.4. Radio Communication Part

Radio frequency means that the carrier power of the frequency can be transmitted through the antenna, and propagates in the free space at the speed of light in the form of alternating electromagnetic fields. When the medium encounters different media, the propagation rate will change, electromagnetic wave reflection and refraction will occur meantime, diffraction. And penetration will cause various losses. It has a skin effect phenomenon when the metal wire is transmitted. Radio frequency technology has a wide and irreplaceable role in the field of wireless communication. The radio frequency communication part transmits and receives high-definition video by directly transmitting and receiving electromagnetic wave signals to achieve communication purposes. By comparing several popular transceiver chips on the market, the nRF905 is finally used, and the whole chip is packaged in QFN, which is only 5 × 5 mm.

## 4. Improved Image Scrambling Algorithm Based on Fibonacci-*p* Coding

### 4.1. Fibonacci-p Transform

Fibonacci-*p* coding is defined as:

(1) $$F_{p}(n)\{\begin{array}{ccc} 0, & & n<1\\ 1, & & n=1\\ F(n-1)+F(n-p-1), & & n>1 \end{array}$$

In the formula, *p* is a non-negative integer. According to the above definition, the Fibonacci-*p* coding sequence will vary depending on the *p*-value. The following special situations are pointed out:

(1) when *p* = 0, the Fibonacci-0 coding sequence is a power sequence of 2 that 1, 2, 4, 8, 16, ...
(2) when *p* = 1, the Fibonacci-1 coding sequence is the classical Fibonacci sequence 1, 1, 2, 3, 5, 8, 13, 21, ...
(3) When *p* > 1, Such as a Table 1.

Suppose *F_p_*(*n*) and *F_p_*(*n* + 1) are two adjacent Fibonacci-*p* coding terms that called the permutation {*T*_1_, *T*_2_, *T*_3_, *...*, *T_Fp_*_(*n*+1)−1_} of the input sequence {1, 2, 3, 4, ..., *T_Fp_*_(*n*+1)−1_} is a one-dimensional Fibonacci-*p* transformation, where {*T*_1_, *T*_2_, *T*_3_, ..., *T_Fp_*_(*n*+1)−1_} is defined as:

(2) $$T_{k}=k[F_{p}(n)+i]modF_{p}(n+1)$$

In the above formula, *k* = 0, 1, ..., *F_p_*(*n* + 1) − 1; *i* = −3, −2, −1, 0, 1, 2, 3; *F_p_*(*n*) + *i* < *F_p_*(*n* + 1).

For example, an *M* × *N* grayscale image whose data is a two-dimensional matrix *A*, expressed as:

(3) $$A=\{\begin{array}{cccc} a_{11} & a_{12} & \ldots & a_{1N}\\ a_{21} & a_{22} & \ldots & a_{2N}\\ \vdots & \vdots & & \vdots \\ a_{M1} & a_{M3} & \ldots & a_{MN} \end{array}$$

*A* column coefficient matrix is generated using Equation (3) according to different *p* values. The image has *N* columns, and the input sequence is *k* = 1, 2, 3, ..., *N*, so *N = F_p_*(*n* + 1) − 1.

For a *p*-value, the output sequence *W(N)* shall be an arrangement of the input sequence {1, 2, 3, ..., *N*}, expressed as:

(4) $$W(N)=(T_{p1},T_{p2},T_{p3},\ldots T_{pN})\;$$

The calculation method of the column coefficient matrix *T_p_*(*N*,*N*) of the two-dimensional Fibonacci-*p* transformation is:

(5) $$T_{p}(i,j)=\{\begin{array}{cc} 1, & \left(T_{pj},j\right)\\ 0, & other \end{array}$$

Similarly, this image matrix has *M* rows, for a p value, the output sequence should be an arrangement of the input sequence {1, 2, 3, ..., *M*}, expressed as:

(6) $$W(M)=(T_{p1},T_{p2},T_{p3},\ldots T_{pM})$$

The calculation method of the row coefficient matrix *T_r_*(*M*,*M*) of the two-dimensional Fibonacci-*p* transformation is:

(7) $$T_{r}(i,j)=\{\begin{array}{cc} 1, & \left(i,T_{pi}\right)\\ 0, & other \end{array}$$

Suppose *B* is the original image matrix, *T_R_* is the row coefficient matrix, and *T_c_* is the column coefficient matrix. Then the following matrix is called the two-dimensional Fibonacci-*p* transform, that is:

(8) $$S=T_{r}BT_{c}$$

In the formula, *S* is a scrambled image matrix which size is *M × N*. The essence is to use the row transformation and the column transformation sequence to perform row-by-row column-by-column scrambling. Firstly, the floating-point chaotic sequence is generated, and then the ascending (or descending) order is arranged to obtain the original sequence in the chaotic state. The sorting sequence is obtained directly. According to the above analysis, when the row and column transformation sequences are obtained, the chaotic transformation can be performed, and the result that Fibonacci-*p* transformation [13,14,15,16,17].

For 256 × 256 images, *N* + 1 = 257, there is:

(9) $$\begin{array}{l} F(16)=189<N+1<F(17)=277,\\ L(16)=249<N+1<L(17)=365 \end{array}$$

Suppose the key *p* = 2, *i* = 1, according to the above formula:

(10) $$T_{f}(k)=k\times [F(16)+1]\mathrm{mod}F(17)=k\times 190\mathrm{mod}277(k=1,2,3,\ldots ,256)\;$$

(11) $$T_{f}(k)=k\times [L(16)+1]\mathrm{mod}L(17)=k\times 250\mathrm{mod}365(k=1,2,3,\ldots ,256)$$

Used for row transform and column transform scrambling sequences, respectively [18,19,20]. The following data is the first 20 numbers of the Fibonacci-2 (*i* = 1) transformation generated according to Equation (10).

Fibonacci-*p* Transformation:

190, 103, 16, 206, 119, 32, 222, 135, 48, 238 151, 65, 254, 167, 80, 183, 96, 9, 199, 112

It is not difficult to verify *k* × 250/356 = *k* × 50/73, so *T*_l_ (73) = 0, *T*_l_ (73 + *k*) = *T*_l_ (*k*), *k* = 1, 2, 3, ...

The above calculation results are consistent with the theoretical analysis, which indicates that when *F_p_*(*n*) + *i* and *F_p_*(*n* + 1) are not mutually prime, it is impossible to generate the arrangement of {1, 2, ..., 256} by using Equation (10) or Equation (11). In fact, the following theorem holds [21]:

(12) $$T_{k}=k\times M\mathrm{mod}N(k=1,2,3,\ldots ,N)$$

The necessary and sufficient condition for the generated set {*T*(1), *T*(2), *T*(3), ..., *T*(*N*)} to be an arrangement of {0, 1, 2, ..., *N −* 1} is *M* and *N* are mutual. If the notation *gcd*(*A*, *B*) is used to represent the greatest common factor of the integers *A* and *B*, the sufficient and necessary condition of the appeal theorem can be expressed as:

(13) gcd(M,N)=1

**Proof:**  (adequacy). We adopt the counter-evidence. Suppose Equation (13) is established, assuming that the set {*T*(1), *T*(2), *T*(3), ..., *T*(*N*)} $generated by Equation (10) is not an arrangement of {0, 1, 2, ..., *N −* 1}, there are two numbers at least, so that *T**(**p**) =** T**(**q**)(**p* < *q* ≤ *N**)**.* Suppose *T*(*p*) = *T*(*q*) = *r*, according to the Equation (12), that is:

$$p•M=p^{\prime }•M+r,\;q•M=q^{\prime }•M+r$$

The meaning of the first expression of the above formula is that *P* is divided by *N* to obtain the divisor *p’* and the remainder *r*, and the meaning of the second expression is similar. The remainder of the two equations is the same. then

(14) $$(q-p)•M=q•M-p•M=q^{\prime }•N-p^{\prime }•N=(q^{\prime }-p^{\prime })•N\;$$

In the equation, the right side of the equal sign indicates that the prime number *N* can be divisible by (*q − p*)*•M*, which can be expressed mathematically as *N*|[(*q − p*)*•M*]. *N*|(*q − p*) can be derived from Equation (13), but 0 < *q* − *p* < *N*, so this is not true. Proof of the end.

(Necessity). Assuming that conditional expression (13) does not hold, there is an integer *d >* 1, so that:

(15) gcd(M,N)=d

Thus, there are positive integers *m* and *n* (*m* < *N*) respectively, so that:

*M* = *m* • *d*, *N* = *n* • *d*

Thus, Equation (12) is:

(16) (q•p)T(k)= k•MmodN=k•(m•d) mod(n•d) 

Because *m* < *n*, it is obtained by Equation (16)

T(1)=m•d

On the other hand, it can be obtained by Equation (16):

$$\begin{array}{ll} T(n+1) & =(n+1)•(m•d)(\mathrm{mod}N)\\ & =n•m•d+m•d(\mathrm{mod}N)\\ & =m•N+m•d(\mathrm{mod}N)\\ & =m•d=T(1) \end{array}$$

Because *n* + 1, we get an arrangement where the set {*T*(1), *T*(2), *T*(3), ..., *T*(*N*)} is not {0, 1, 2, ..., *N* − 1}. Proof of the end.

Suppose *S* is a scrambling image matrix, *TR* is a row coefficient matrix, and *Tc* is a column coefficient matrix. The following matrix is called a two-dimensional inverse p-Fibonacci transform, that is:

(17) $$R=T_{r}{}^{-1}ST_{c}{}^{-1}$$

In the formula, *R* is a reconstructed image matrix.

### 4.2. Color Space and Its Conversion

*YCbCr* is a common color space, the common JPEG image format usually uses this color space, which is derived from the YUV color space adopted by the European TV system, where *Y* represents transparency, and *Cb* and *Cr* are the components obtained by making a small adjustment of *U* and *V* [22]:

(18) $$\left[\begin{array}{c} Y\\ Cb\\ Cr\\ 1 \end{array}\right]=\left[\begin{array}{cccc} 0.2290 & 0.5870 & 0.1140 & 0\\ -0.1687 & -0.3313 & 0.5000 & 128\\ 0.5000 & -0.4187 & -0.0813 & 128\\ 0 & 0 & 0 & 1 \end{array}\right]\left[\begin{array}{c} R\\ G\\ B\\ 1 \end{array}\right]$$

and the inverse conversion formula is:

(19) $$\left[\begin{array}{c} R\\ G\\ B \end{array}\right]=\left[\begin{array}{ccc} 1 & 1.40200 & 0\\ 1 & -0.34414 & -0.71414\\ 1 & 1.77200 & 0 \end{array}\right]\left[\begin{array}{c} Y\\ Cb-128\\ Cr-128 \end{array}\right]$$

### 4.3. Improved Image Scrambling Algorithm

In this paper, the algorithm applies two-dimensional Fibonacci-*p* transform DCT coefficients and symbols. It is proven that proposed algorithm is a lossless scrambling algorithm.

The steps of the image scrambling algorithm are as follows:

**Input**: Color or grayscale image that needs to be scrambled.

**Output**: Scrambling color images or scrambling grayscale images.

**Step--1**: Select the key parameter *P* to calculate the matrix row and column coefficient matrix of the two-dimensional Fibonacci-*p* transform.

**Step--2**: Convert the color image into *YCbCr* form and divide it into *Y*, *Cb* and *Cr* those three components. Each component is a two-dimensional matrix (the grayscale image omits this step).

**Step--3**: Transform each component into the frequency domain using a DCT transform (the grayscale image is applied to the DCT transform directly).

**Step--4**: The DCT domain data matrix of each component is scrambled using a two-dimensional Fibonacci-*p* transform.

**Step--5**: Separate the positive and negative symbols and the other size values of the Scrambled DCT domain data into two matrices (the grayscale image separates the positive and negative symbols and their size values into two matrices directly).

**Step--6**: The two-dimensional Fibonacci-*p* transform is used to scramble the positive and negative symbol matrices of each color component DCT domain again that to obtain a scrambled symbol matrix, (the grayscale image directly applies the two-dimensional Fibonacci-*p* transform to the symbol matrix of the DCT domain to obtain a scrambled symbol matrix).

**Step--7**: Combine the scrambled symbol matrix with the data matrix to obtain a *YCbCr* image and convert it into RGB scrambled color image (the grayscale image scrambles the DCT domain symbol matrix and its value Matrix synthesis directly then get scrambled images).

The steps of disarming the frequency domain scrambling algorithm are the following:

**Input**: Scrambled color or grayscale images.

**Output**: Reconstruct color image or scramble grayscale image.

**Step--1**: Calculate the inverse matrix row and column coefficient matrix of the two-dimensional Fibonacci-*p* transform by using the keys *p* and *i*.

**Step--2**: Convert the color scrambled image into *YCbCr* form and divide it into three components *Y*, *Cb*, and *Cr*. Each component is a two-dimensional matrix (the gray image separates the positive and negative symbols and their size values those two matrices directly).

**Step--3**: Transform the positive and negative symbols matrix of the DCT domain data of each component with a two-dimensional inverse Fibonacci-*p* transform, and obtain a reconstructed symbol matrix of three components (the positive and negative symbol matrix of the gray image is transformed by a two-dimensional inverse Fibonacci-*p* directly and the reconstructed symbol matrix is obtained).

**Step--4**: Combine the reconstructed symbol matrix and the numerical matrix of the three color components (combine reconstructed symbol matrices and numerical matrices directly in grayscale images).

**Step--5**: The two-dimensional inverse Fibonacci-*p* transform is performed on the synthetic matrix obtained in step 4.

**Step--6**: Perform the DCT inverse transform on the matrix obtained in step 5 (the grayscale image performs the DCT inverse transform on the grayscale image obtained).

**Step--7**: Combine the three reconstructed color components to obtain a *YCbCr* image and convert it into an RGB image (the grayscale image omits this step).

## 5. Experimental Results and Analysis

### 5.1. Correlation Analysis of Adjacent Pixels

*Calculating Image Adjacent Pixel Correlation*. The individual pixels in the digital image do not exist independently, and the correlation between the pixels is large, which means that there is a small difference in the gray value in a large area of the image. One of the goals of an encrypted image is to reduce the correlation of adjacent pixels, which mainly includes the correlation between horizontal pixels, vertical pixels, and diagonal pixels. The smaller the correlation, the better the encryption effect and the higher the security:

(20) $$r_{xy}=\frac{cov(x,y)}{\sqrt{D(y)}\sqrt{D(x)}}$$

(21) $$D(x)=\frac{1}{N}\sum_{i=1}^{N}(x_{i}-E(x){)}^{2}\;$$

(22) $$cov(x,y)=\frac{1}{N}\sum_{i=1}^{N}(x_{i}-E(x))(y_{i}-E(x))$$

In the formula, *x* and *y* are the gray values of two adjacent pixels in the image. *E* (.), *D* (.) and *Cov* (.) are the expectation, variance and covariance respectively, *r* is the correlation coefficient of adjacent two pixels. The higher the value of its value is close to 1, the higher the correlation of adjacent pixels. If images were encrypted by original scrambling encryption algorithm and improved scrambling encryption algorithm respectively, then compare the correlation between adjacent pixels in 3 directions.

**Figure 2:**
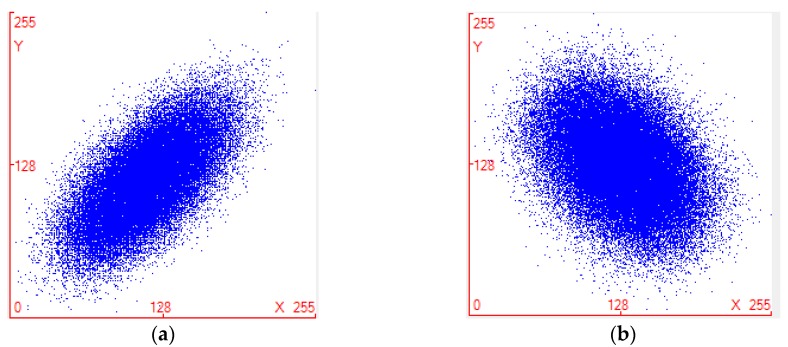
(**a**) Correlation distribution diagram of image scrambling encryption algorithm; (**b**) Correlation distribution of improved image scrambling encryption algorithm.

Figure 2a,b respectively show the correlation distribution of two horizontally adjacent pixels in the original algorithm and the improved algorithm encryption image. Figure 2b is more visually dispersed than the correlation distribution of Figure 2a, from Figure 2b with the comparison of original correlation icon shows that the correlation between the original algorithm encrypted image and the improved encrypted image is completely separate. The analysis of the degree of correlation image shows that the proposed algorithm is superior to the original scrambling encryption algorithm.

### 5.2. Histogram Analysis of Encrypted Image

The variance is used to evaluate the consistency of histogram distribution and indicate the degree of dispersion between the histogram and its average value, the consistency of the distribution is expressed by the size of variance value. The smaller the variance the more uniform distribution. The histogram of image is represented by *hist_i_*, and the formula of variance is:

(23) $$S=\frac{1}{256}\sum_{i=0}^{255}{\left(hist_{i}-aver\right)}^{2}$$

And the average (aver) is:

(24) $$aver=\frac{1}{256}\sum_{i=0}^{255}hist_{i}$$

**Figure 3:**
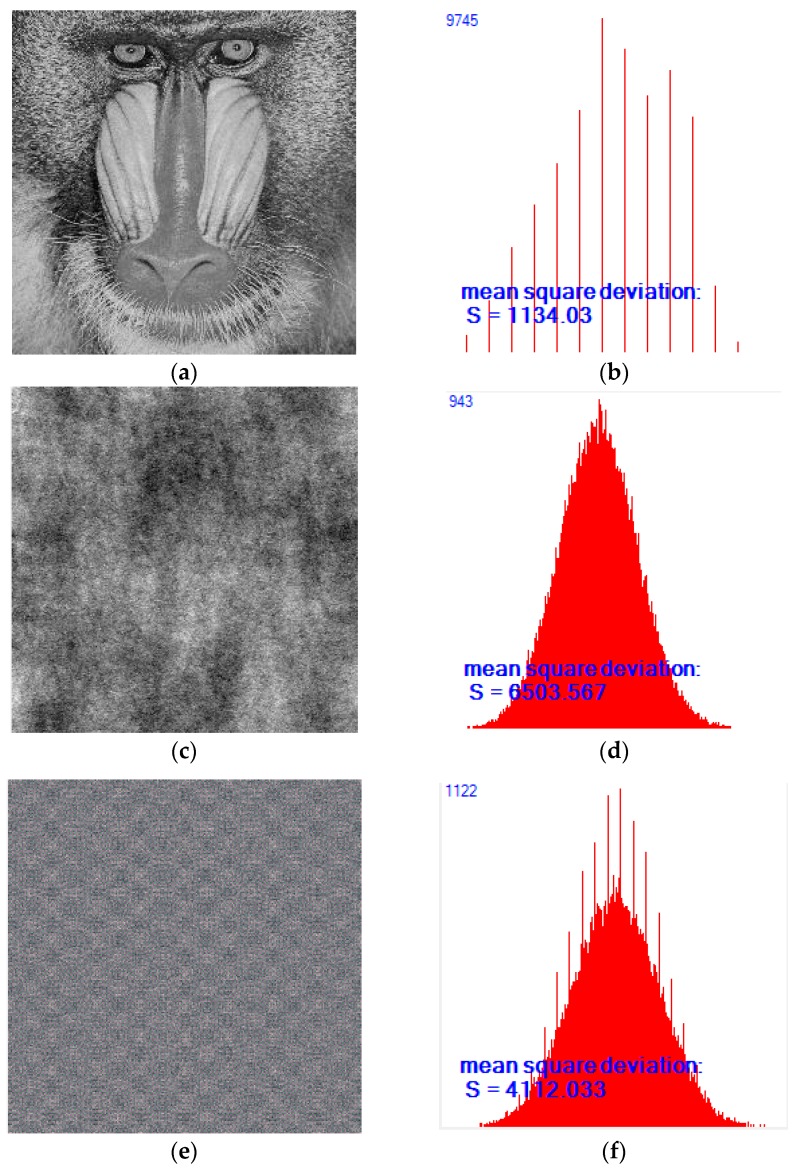
(**a**) Original image; (**b**) Histogram of original image; (**c**) Original image scrambling encryption image; (**d**) Histogram of image scrambling encryption image; (**e**) Improved image scrambling encryption image; (**f**) Histogram of improved image scrambling encryption image.

If the pixel value can be distributed evenly that in the range of (0–255) after encryption, the uniform distribution of gray histogram will be regarded as the ideal state. From the above three images, it is found that the histogram (Figure 3b) of the original image (Figure 3a) is uneven, the uniformity of distribution is not ideal, the distribution effect (Figure 3d) of the traditional image scrambling encryption image (Figure 3c) has not been improved obviously, which has poor performance, and the pixel correlation of the image is not weakened. Compared with the traditional image scrambling encryption histogram, the histogram (Figure 3f) of the improved algorithm encryption image (Figure 3e) is more concentrated and gentler. The value of the variance is obviously smaller than variance of the traditional Hill encryption algorithm, which weakens the correlation greatly and it is result is ideal.

### 5.3. Information Entropy and Peak Signal to Noise Ratio

*Information Entropy* is a concept that used to measure the amount of information in information theory, which contains information content of an image, the system is more orderly that information entropy is lower. The information entropy of the image is:

(25) $$H(m)=-\sum_{i=0}^{L-1}p(m_{i})log_{2}p(m_{i}),\sum_{i=0}^{L-1}p(m_{i})=0$$

where *L* and *m_i_* indicate that gray value is *m_i_* and description is *L*, *P_mi_* indicates the probability of the appearance of the gray value. When the probability of gray value appears in the image is equally, it is largest that information entropy of the image, and its gray distribution is identical. When information entropy is equal to 8 that is proved the random distribution of images is more ideal.

The *Peak Signal-to-Noise Ratio* is used to estimate the distortion of some lossy encryption. *PNSR* reflects the quality of the encryption algorithm, which indicates the change between the original picture and the encrypted picture. Its calculation formula is:

(26) $$PSNR=10\times lg\left[\frac{M\times N\times 255^{2}}{\sum_{i=0}^{M-1}\sum_{j=0}^{N-1}{\left(p(i,j)-c(i,j)\right)}^{2}}\right]$$

In the formula, *M* and *N* represent the width and height of the image, and *p*(*i,j*) and *c*(*i,j*) are the pixel values of the original image and the decrypted image, respectively, and the smaller the value, the better the encryption quality. When *p*(*i,j*) and *C*(*i,j*) are the original picture and the decryption picture, respectively, the *PSNR* reflects the distortion of the encryption algorithm on the other hand. The larger the value, the smaller the algorithm’s amount distortion, and the encrypted the higher the quality [23,24,25,26,27,28,29].

*Diffusion* is an important feature in the encryption algorithm and as proposed by Shannon [30], an excellent encryption system must have good diffusivity. The meaning is that when a bit is changed in the original image, the encryption image will be changed in an unpredictable way. The diffusivity of the image encryption algorithm indicates that the output pixels of the encrypted image should be dependent on the input pixels of the original image in a very complicated way, which can resist the attacker’s analysis of the algorithm. Attackers usually make small changes to the original image, and then use the algorithms used as attackers to encrypt the original and modified images, and compare the relationship between the original and the encrypted images by comparing two images. This kind of attack becomes a difference attack [31,32,33]. One pixel of the original image is modified by the attacker, looking at the changes in the result, it is possible for attacker to find a relationship between the original image and the encrypted image. If a small change in the original image can cause significant changes in the effects of diffusion and chaos, the efficiency of the differential attack is very low and the attack is invalid. In order to verify the influence of a pixel change in the entire encrypted image, two measurement methods are commonly used one is pixel change rate and the other is uniform average change intensity. Two encrypted images are represented by *C*1 and *C*2 respectively, only one pixel is different in their corresponding original images, the gray values of images *C*1 and *C*2 at coordinates (*i*,*j*) are represented by *C*1(*i*,*j*) and *C*2(*i*,*j*), respectively. The uniform average change intensity UACI is defined as:

(27) $$UACI=\frac{\sum_{i=0}^{M-1}\sum_{j=0}^{N-1}\left|C_{1}(i,j)-C_{1}(i,j)\right|}{255\times M\times N}\times 100\%$$

From Table 2, we can know the information entropy of the original image is equal to 3, the entropy of the traditional image scrambling encryption image is about 6. It is shown that the traditional image scrambling encryption algorithm does not make a significant change in the probability of the random distribution of the image, and there is no more agreement on the gray distribution. Information entropy is bigger that the image is more orderly and the probability of the image random distribution is smaller. Using the improved encryption algorithm to encrypt image, the information entropy is increased from the original data to 7, the information entropy is greater, random distribution of the image is more ideal, the more consistent in the grey distribution and the encryption effect is more ideal. From the pixel change rate, we can learn that the results of the improved algorithm are larger than the traditional image scrambling algorithm on the numerical value, and the results of the uniform average change intensity have a little difference [34,35,36]. It is clear that the original image has no/zero resistance against differential attack. When we compare the proposed scheme with the scheme proposed in traditional, our proposed scheme has higher values of UACI indicating high resistance against different attacks.

### 5.4. Time Analysis

An efficient algorithm should use minimum resources and minimum computation cost. To check the computational complexity, we have outlined the results of the proposed scheme is compared with original schemes. Table 3 shows time taken during encryption of plaintext images. As decryption is the reverse process of encryption, the decryption time is almost the same as the encryption time. It is highlighted from Table 3 that the proposed scheme has less computational complexity when compared with original schemes.

## 6. Conclusions

In this paper, the tool used in this experiment is Visual Studio 2015, using the c.NET language. We introduced a video wireless transmission for UAVs and the module of the system briefly, aiming at the favorable security and efficient efficiency of traditional image encryption technology, an improved image scrambling encryption algorithm based on Fibonacci-*p* coding. The first new idea of the algorithm is to separate the positive and negative signs and data of the scrambled DCT coefficients, then form the symbol matrix and the data matrix respectively [37,38], perform the scrambling encryption operation on the symbol matrix. The second new idea is to encrypt the color RGB image by converting the *R*, *G*, and *B* colors into *Y*, *Cb*, and *Cr*, and converting the normal image operation into operations on *Y*, *Cb*, and *Cr*, thereby implementing the encryption operation. Results of experiments show that the algorithm has high efficiency of scrambling and the disorderly effect is uniform and the correlation of adjacent pixels is small, which changes the statistical information of the image and that is more ideal in the random distribution of the image and gray level.

Through the analysis of its performance theory and experimental results, it has been shown that the improved algorithm is more successful than traditional image scrambling encryption algorithms and has great developmental potential. From the analysis of the degree of influence of variance and peak signal-to-noise ratio change, the algorithm does not significantly increase the variance value, the value has been improved compared to the original algorithm but still does not achieve the desired range. Therefore, considering the improvement of this aspect will be the future direction.

## Figures and Tables

**Table 1:** Coding sequences corresponding to different *p* values.

	*n*	1	2	3	4	5	6	7	8	…
*p*										
0		1	2	4	8	16	32	64	128	…
1		1	1	2	3	5	8	13	21	…
2		1	1	1	2	3	4	6	9	…
3		1	1	1	1	2	3	4	5	…
…		…	…	…	…	…	…	…	…	…
∞		1	1	1	1	1	1	1	1	…

**Table 2:** Analysis of Test Results.

Information	Artwork	Original Algorithm	Article Algorithm
Entropy	3.362699	6.864638	7.045538
PSNR	0.00	10.835432	13.287045
UACI	0.00	23.398628	15.736912

**Table 3:** Time (ms) taken during encryption.

Image	Original Algorithm	Article Algorithm
Baboo	3059.2593	898.0163
Camaraman	2589.3521	988.4307
Boats		885.9098

## Appendix A

The other two images that were used:
